# Prevalence of and Associated Factors for Overactive Bladder Subtypes in Middle-Aged Women: A Cross-Sectional Study

**DOI:** 10.3390/medicina58030383

**Published:** 2022-03-04

**Authors:** Cheng-Fang Yang, Chao-Yuan Huang, Shu-Yi Wang, Shiow-Ru Chang

**Affiliations:** 1 Second Degree Bachelor of Science, National Taiwan University, Taipei 100, Taiwan; fionayang@ntu.edu.tw; 2 Department of Nursing, National Taiwan University Hospital, Yunlin Branch, Yunlin 640, Taiwan; 3 Department of Urology, National Taiwan University, Taipei 100, Taiwan; cyhuang0909@ntu.edu.tw; 4 Department of Urology, National Taiwan University Hospital, Taipei 100, Taiwan; 5 Loretto Heights School of Nursing, Regis University, Denver, CO 80221, USA; swang@regis.edu; 6 School of Nursing, National Taiwan University, Taipei 100, Taiwan; 7 Department of Nursing, National Taiwan University Hospital, Taipei 100, Taiwan

**Keywords:** prevalence, middle-aged, risk factor, overactive bladder, urinary incontinence

## Abstract

*Background and Objectives*: The living environment can manifest physiological responses in humans, with cohabiting couples often having similar health statuses. The aim of this study was to (1) examine the prevalence of the overactive bladder (OAB) with or without incontinence and (2) identify associated factors for OAB with and without incontinence (including environmental factors, such as living with a partner who has OAB) in middle-aged women. *Materials and Methods*: In this cross-sectional study, the International Consultation on Incontinence Questionnaire Overactive Bladder (ICIQ-OBA) was administered to 970 couples. Data were analyzed using descriptive statistics, chi-square analyses, and multivariate logistic regression. *Results*: Responses to the ICIQ-OBA among middle-aged women generated a higher prevalence of OAB with incontinence (OAB_wet_; 41%) than OAB without incontinence (OAB_dry_; 26%; *p* < 0.001). The factors associated with OAB_wet_ were as follows: being age ≥ 55 years (odds ratio [OR], 1.41; 95% confidence interval [CI], 1.02–1.95), having a body mass index (BMI) ≥ 27 kg/m^2^ (OR, 1.50; 95% CI, 1.03–2.17), having vaginitis (OR, 1.89; 95% CI, 1.28–2.80), and having partners with OAB_wet_ (OR, 2.35; 95% CI, 1.74–3.19). Having partners with OAB_dry_ (OR, 1.81; 95% CI, 1.34–2.44) was an associated factor for OAB_dry_. *Conclusions*: This study identified the associated factors for OAB subtypes (OAB_wet_ and OAB_dry_) in middle-aged women. These findings can support treatment and preventive strategies for health providers who care for patients with OAB. As part of the treatment and preventative strategies, the risk that partners may introduce to the development of OAB in women should also be considered.

## 1. Introduction

Individuals tend to pursue less social interaction and have a poorer quality of life when they have symptoms of an overactive bladder (OAB) [1,2,3,4]. Studies have demonstrated that OAB prevalence varies greatly among women aged ≥ 18 years, ranging from approximately 17% to 43% in Europe and the United States [2,5,6,7,8] and from approximately 1.9% to 53.8% in Asia [1,3,4,9,10]. Studies have indicated that rates of OAB without incontinence (OAB_dry_) and with incontinence (OAB_wet_) range from 2.4% to 10.3% and from 2.0% to 9.3%, respectively [1,7,8,11]. The prevalence of OAB and its subtypes increases with age and is a common problem among middle-aged adults (aged ≥ 40 years), with more women (11–39.5%) experiencing this disorder than men (11–27%) [1,2]. To date, few studies have investigated the prevalence of OAB_dry_ and OAB_wet_ in middle-aged women.

Previous studies have identified the following factors affecting OAB incidence: age [1,8,9,12,13,14], education level [1], employment [1], prior disease [6,9,10,15,16], parity [1,11], mode of delivery during birth [1,11], high body mass index (BMI) [6,7,9,11,13], racial or ethnic identity [6], geographical region [1], dietary habits [1,14,17], and environmental stimuli [14,18]. By contrast, one study found that sex, educational level, parity, vaginal delivery, race, menopause, marital status, smoking and alcohol consumption were not associated with OAB [13]. One study reported that the main factor influencing immunological variation in humans was cohabitation [19]. Cohabiting couples often have similar or concordant health statuses that tend to converge over time [20]. Their daily life activities are intertwined, and their personal attributes, such as lifestyle, affect each other, which may contribute to behavioral convergence [21]. Additionally, Kiecolt-Glaser argued that cohabiting couples, although not genetically related, share a common living environment, pool resources, consume food together, and share a social network. This concordance may influence their health and health behaviors [22]. Few studies have demonstrated that sex-specific demographic characteristics and patterns of life are associated with OAB_dry_ and OAB_wet_ [1]. Additionally, no studies have evaluated the relationship between cohabiting with a partner with OAB and OAB in middle-aged adults. Therefore, the aim of this study was twofold: (1) to examine the prevalence of OAB_dry_ and OAB_wet_ and (2) to identify environmental (e.g., having a cohabitating partner with OAB) and other risk factors for OAB_dry_ and OAB_wet_ in middle-aged women.

## 2. Materials and Methods

### 2.1. Study Design and Setting

A cross-sectional design and convenience sampling were conducted in three communities of northern cities in Taiwan. This research project was reviewed and approved by the Research Ethics Committee. The inclusion criteria were (1) middle-aged women (40–65 years) with cohabiting partners who also agreed to participate and (2) the ability of the women and their partners to complete the questionnaires themselves. Participant recruitment was generated by undergraduate students and consisted of their parents. The first author approached undergraduate students at three universities and explained the research purpose, asking the students to pass along a research pamphlet to their parents requesting their participation in the study. Several documents (informed consent, instructions, a demographic and medical characteristics form, and questionnaires) were subsequently mailed to participants. When the participants had completed the documents, they returned them to the researchers using a stamped envelope.

### 2.2. Measurements

The International Consultation on Incontinence Questionnaire Overactive Bladder (ICIQ-OAB) [23] was used in this study. This scale was designed to assess urinary frequency, nocturia, urgency and the presence of urgent urinary incontinence within the past four weeks on a 5-point Likert scale. The questionnaire content was translated from English to Chinese and then back-translated from Chinese to English. The test–retest reliability over a 4-week interval was 0.8 (*p* < 0.05). Five experts reported a content validity of 1.0 for the Taiwanese population. The internal consistency of Cronbach’s *α* was 0.86 for the participants. Frequency, urgency and urgent incontinence scores of >0 and a nocturia score of >1 indicated probable OAB. Nocturia, in this study, referred to waking up at night at least twice to urinate, a definition adopted by other studies [7,8,11,15,24], and OAB was subcategorized into OAB_dry_ and OAB_wet_ [1,8,11].

The four scored items of the ICIQ-OAB are as follows: (1) “How often do you pass urine during the day?” (frequency); (2) “During the night, how many times do you have to get up to urinate, on average?” (nocturia); (3) “Do you have to rush to the toilet to urinate?” (urgency); and (4) “Does urine leak before you can get to the toilet?” (urge urinary incontinence). Each item is followed by the question, “How much does this bother you?” that was scored on a visual analog scale from 0 (not at all) to 10 (a great deal); this assisted in understanding the extent to which specific symptoms affected the respondents psychologically, but these scores were not included in the analysis.

The following symptom combinations constituted the criteria for subcategorization:

OAB_dry_: urgency and frequency, (or) urgency and nocturia, (or) urgency and frequency and nocturia.

OAB_wet_: urgency and urge incontinence; (or) urgency, frequency and urge incontinence; urgency, nocturia and urge incontinence; (or) urgency, frequency, nocturia and urge incontinence.

### 2.3. Data Analysis

Descriptive statistics were used to summarize data on demographic and disease characteristics. Additionally, data were presented in terms of frequency and percentage. The chi-square test was performed to assess the associations between the two OAB subtypes and demographic characteristics (e.g., age, marital status, education level, employment status, income, BMI, smoking, drinking and exercise status, and cohabitation with a partner), and medical history (including their disease history and present illnesses), and gynecological diseases. To identify associated factors, multivariate logistic regression with backward elimination was used to calculate the odds ratios (ORs) and 95% CI. The multivariate model included variables with *p* < 0.1 in the chi-square test. A *p* value < 0.05 indicated significance.

## 3. Results

### 3.1. Participant Characteristics and Their Associations with OAB_dry_ and OAB_wet_

A total of 1090 couples were enrolled in this study, with 120 couples excluded due to incomplete data, them falling outside the age group targeted by this study, or them not living together. Thus, 970 couples (89%) were included in the analysis. The overall prevalence of OAB was 67% in middle-aged women, and among them, the prevalence of OAB_wet_ (41%) was significantly higher than that of OAB_dry_ (26%; *p* < 0.001; Table 1).

First, having a partner with OAB_dry_ was significantly associated with having OAB_dry_ (*p* < 0.001). Next, age (*p* = 0.008), BMI (*p* = 0.039), parity (*p* = 0.084), mode of delivery (*p* = 0.085), having a partner with OAB_dry_ (*p* = 0.086), and having a partner with OAB_wet_ (*P* < 0.001) were associated with having OAB_wet_. Although no variables for disease or disorders were associated with OAB_dry_, osteoporosis (*p* = 0.047), arthritis (*p* = 0.027), depression (*p* = 0.083), vaginitis (*p* = 0.001), pelvic inflammation (*p* = 0.012), uterine prolapse (*p* = 0.012), and cervical cancer (*p* = 0.078) were associated with OAB_wet_ in middle-aged women (Table 1). These variables (*p* < 0.1) were treated as covariates or potential factors in the multivariate logistic regression, which was used to verify whether the variables were associated factors for OAB_dry_ and OAB_wet_ in middle-aged women.

### 3.2. Associated Factors for OAB with and without Incontinence in Middle-Aged Women

Table 2 lists the significant factors, identified through multivariate regression, of OAB_dry_ prevalence in middle-aged women. Women who had partners with OAB_dry_ (OR = 1.81; 95% CI: 1.34–2.44) had a related risk of OAB_dry_. The significant associated factors of OAB_wet_ were women aged 55 years or older (OR = 1.41; 95% CI: 1.02–1.95), having a BMI ≥ 27 (OR = 1.50; 95% CI: 1.03–2.17), having a partner with OAB_wet_ (OR = 2.35; 95% CI: 1.74–3.19), and having vaginitis (OR = 1.89; 95% CI: 1.28–2.80; Table 3).

## 4. Discussion

In the present study, we focused specifically on the prevalence of OAB subtypes and their significant associated factors. This study collected data on the prevalence of OAB, OAB_dry_ and OAB_wet_ (67%, 25%, 41%, respectively); however, other studies have calculated the prevalence of OAB, OAB_dry_ and OAB_wet_ in women to be 1.9–54% [1,2,3,4,9,10], 2.4–10.3% [1,8,11], and 2–6.5% [1,8,11], respectively. The inconsistency in results may be due to differences in the age distributions [1,8,9,14], race or ethnicity [6], and/or differences in survey methodology [25]. In other studies, conducting in-person [9] or telephone interviews [2,3,26] was found to easily result in underestimation [25], possibly because participants may have felt reluctant or embarrassed to address such sensitive topics. In contrast to studies that conducted interviews, our study administered a self-reported questionnaire.

In our multivariate logistic models, age, BMI, vaginitis, and having a partner with OAB_wet_ were associated factors for OAB_wet_ in middle-aged women. Additionally, we found that women older than 55 years exhibited an increased prevalence of OAB_wet_. A similar study indicated that the prevalence of OAB_wet_ increases in women aged 50 years or older [1]. Other studies demonstrated that advanced age was also an associated factor for OAB, but they did not distinguish between OAB_dry_ and OAB_wet_ [8,9,12,13,14].

This study found that the prevalence of OAB_wet_ in middle-aged women with BMI ≥ 27 kg/m^2^ was 1.5 times higher than that of those with BMI < 24 kg/m^2^. This finding aligned with a study that found that, in women, higher BMIs were associated with higher OAB_wet_ [7]. In contrast with our study, another study demonstrated that BMI is not associated with OAB_wet_ in women [1]. It may be related to different BMI classifications and the percentage of women with BMI ≥ 28 kg/m^2^ was lower (5.4%) than the study (15.7%). Higher BMI may expose the pelvic floor to increased intra-abdominal pressure and intravesical pressure which may chronically extend the pudendal nerve, leading to nerve injury and pelvic floor dysfunction [27,28] and thus contribute to the development of stress urinary incontinence [27,28] and urge urinary incontinence [28]. However, the OAB symptoms were self-reported by participants in this study rather than clinician diagnosis and were not used to further sort the subtypes of incontinence. Most studies have indicated that BMI has a significant positive association with OAB [6,9,11,13]; however, they did not differentiate between the OAB subtypes of OAB_dry_ and OAB_wet_.

Our study also found that, for middle-aged women, vaginitis was an associated factor for OAB_wet_. For example, patients diagnosed as having OAB had a higher prevalence of comorbid urinary tract infection (UTI) and vulvovaginitis [29], and women with bacterial vaginosis had an increased risk of UTI [30]. This finding is similar to that of a study which found that patients with OAB are associated with vaginitis [16]. Additionally, most studies have indicated a positive association between diabetes and OAB [9,10,15]. However, no association between diabetes and OAB was identified in this study, which may be because few women in our study had diabetes and more than half of the participants had a BMI < 24 kg/m^2^. People with BMI < 25 kg/m^2^ are less likely to have diabetes mellitus and OAB than people with higher BMIs [9].

In this study, having partners with OAB_dry_ and OAB_wet_ placed women at an increased risk of having OAB_dry_ and OAB_wet_, respectively. No prior studies have analyzed whether having a partner with either OAB_dry_ or OAB_wet_ is a factor for OAB in middle-aged women. However, one study revealed that environmental cues, such as another person mentioning going to the bathroom, may constitute Pavlovian-conditioned stimuli that affect OAB symptoms [18]. Studies have revealed that the environmental factors, including dietary habits [1,14,17] and alcohol consumption [1,14], with each is associated with OAB [1,14,17], OAB_dry_ [1], or OAB_wet_ [1]. In this study, all partners lived together and thus shared a living environment, which may have influenced their respective health and health behaviors [22]. Thus, partners might have not only a psychological influence on each other but also influence each other as stimuli within their environment. Although this study is limited by the unclear etiology of this association, environmental factors show strong causation for increasing OAB prevalence; therefore, further investigation is warranted.

This study has several limitations. First, individuals with this study’s OAB subtypes did not undergo any clinical diagnosis or urodynamic testing, but the questionnaire examination of OAB symptoms was reliable and valid [23]. Second, the effects of the factors of having cohabiting partners with or without OAB may be affected by the length of cohabitation and the couple’s demographic characteristics. This, and the underlying mechanism, should be investigated in future studies. Third, due to time constraints and a lack of manpower, a cross-sectional study was performed instead of a longitudinal study.

## 5. Conclusions

This study identified associated factors for OAB_dry_ and OAB_wet_ in middle-aged women. We found a higher prevalence of OAB than that reported by most studies. Additionally, we determined that the associated factors for OAB_wet_ in middle-aged women included age ≥ 55 years, BMI ≥ 27 kg/m^2^, having vaginitis, and having a partner with OAB_wet_. Furthermore, having a partner with OAB_dry_ was an associated factor for OAB_dry_ in middle-aged women. Our study has practical implications for healthcare providers in that it clarifies the factors associated with the occurrence of OAB subtypes and provides valuable information regarding an association between having OAB and cohabiting with a partner who has OAB. When caring for OAB patients, healthcare providers can take the partner factor into account and offer appropriate nursing. In addition, this study revealed that advanced age, BMI ≥ 27 kg/m^2^, and vaginitis were associated factors for the OAB subtypes; therefore, providing education and community outreach activities (e.g., screening) to improve symptom recognition and early diagnosis is essential. The influence mechanism of environmental factors on OAB symptoms merits further examination.

## Figures and Tables

**Table 1:** Participant characteristics and their associations with OAB_dry_ and OAB_wet_.

Variable	Total *n* (%)	OAB_dry_ *n* (%)	*P*	OAB_wet_ *n* (%)	*P*
	970 (100)	248 (25.6)		400 (41.2)	
Age group (year)			0.264		0.008 **
<55	770 (79.4)	203 (81.9)		301 (75.2)	
≥55	200 (20.6)	45 (18.1)		99 (24.8)	
Education level			0.577		0.528
Senior high school or less	651 (67.1)	170 (68.5)		273 (68.2)	
College and above	319 (32.9)	78 (31.5)		127 (31.8)	
Marital status			0.322		0.889
Married	940 (96.9)	238 (96.0)		388 (97.0)	
Non-married	30 (3.1)	10 (4.0)		12 (3.0)	
Employment			0.593		0.123
Full time	557 (57.4)	146 (58.9)		218 (54.5)	
Not full time	413 (42.6)	102 (41.1)		182 (45.5)	
BMI, kg/m^2^			0.242		0.039 *
<24.0	592 (61.0)	151 (60.9)		240 (60.0)	
24.0–26.9	226 (23.3)	65 (26.2)		84 (21.0)	
≥27.0	152 (15.7)	32 (12.9)		76 (19.0)	
Drinking			0.484		0.957
Yes	36 (3.7)	11 (4.4)		15 (3.8)	
Smoking			0.818		0.898
Yes	52 (5.4)	14 (5.6)		21 (5.2)	
Exercise			0.384		0.983
Yes	723 (74.5)	190 (76.6)		298 (74.5)	
Parity			0.381		0.084
None	39 (4.0)	7 (2.8)		14 (3.5)	
1	104 (10.8)	32 (12.9)		32 (8.0)	
2	464 (47.8)	121 (48.8)		193 (48.2)	
≥3	363 (37.4)	88 (35.5)		161 (40.3)	
Mode of delivery			0.128		0.085
None	26 (2.7)	4 (1.6)		7 (1.8)	
Exclusive vaginal	584 (60.2)	139 (56.0)		256 (64.0)	
Exclusive cesarean	262 (27.0)	73 (29.5)		95 (23.8)	
Vaginal delivery + cesarean	98 (10.1)	32 (12.9)		42 (10.4)	
Menstrual status			0.447		0.529
Premenopause	668 (68.9)	166 (66.9)		271 (67.8)	
Menopause	302 (31.1)	82 (33.1)		129 (32.3)	
Partner OAB_dry_			<0.001 **		0.086
Yes	326 (33.6)	108 (43.5)		122 (30.5)	
Partner OAB_wet_			0.076		<0.001 **
Yes	236 (24.3)	50 (20.2)		136 (34.0)	
Medical disease					
Diabetes Mellitus			0.902		0.284
Yes	34 (3.5)	9 (3.6)		11 (2.8)	
Hypertension			0.494		0.189
Yes	106 (10.9)	30 (12.1)		50 (12.5)	
Cardiovascular disease			0.385		0.411
Yes	31 (3.2)	10 (4.0)		15 (3.8)	
Osteoporosis			0.464		0.047 *
Yes	77 (7.9)	17 (6.9)		40 (10.0)	
Arthritis			0.920		0.027 *
Yes	56 (5.8)	14 (5.6)		31 (7.8)	
Depression			0.343		0.083
Yes	28 (2.9)	5 (2.0)		16 (4.0)	
Cancer			0.166		0.958
Yes	27 (2.8)	10 (4.0)		11 (2.8)	
Gynecologic disease					
Vaginitis			0.384		0.001 **
Yes	125 (12.9)	28 (11.3)		69 (17.2)	
Pelvic inflammatory			0.165		0.012 *
Yes	28 (2.9)	4 (1.6)		18 (4.5)	
Myoma uterine			0.623		0.175
Yes	170 (17.5)	46 (18.5)		78 (19.5)	
Uterine Prolapse			0.685		0.012 *
Yes	10 (1.0)	2 (0.8)		8 (2.0)	
Cervical cancer			0.189		0.078
Yes	5 (0.5)	0 (0.0)		4 (1.0)	
Ovary cancer			0.407		0.801
Yes	2 (0.2)	0 (0.0)		1 (0.2)	
Hysterectomy			0.754		0.196
Yes	70 (7.2)	19 (7.7)		34 (8.5)	
Oophorectomy			0.178		0.437
Yes	54 (5.6)	18 (7.3)		25 (6.2)	

OAB_dry_, overactive bladder without urinary incontinence; OAB_wet_, overactive bladder with urinary incontinence; BMI, body mass index. The associations between participant characteristics and OAB_dry_ or OAB_wet_ were examined using chi-square analyses (*P* < 0.1); significant covariates were used in a subsequent multivariate regression model; * *P* < 0.05; ** *P* < 0.01.

**Table 2:** Associated factors for OAB_dry_ in middle-aged women.

Variables	β	OR (95% CI)	*P*
Partner OAB_dry_			
Yes	0.59	1.81 (1.34–2.44)	<0.001 **
Partner OAB_wet_			
Yes	−0.47	0.95 (0.65–1.41)	0.815

OAB_dry_, overactive bladder without urinary incontinence; OAB_wet_, overactive bladder with urinary incontinence. The multivariate logistic regression analysis included variables with *P* < 0.1 for OAB_dry_ in chi-square analysis, including having the variable of having a partner OAB_dry_ and OAB_wet_; ** *P* < 0.01.

**Table 3:** Associated factors for the OAB_wet_ in middle-aged women.

Variables	β	OR (95% CI)	*P*
Age group (yr)			
<55	Ref.		
≥55	0.35	1.41 (1.02–1.95)	0.036 *
BMI, kg/m^2^			
<24.0	Ref.		
24.0–26.9	−0.13	0.88 (0.64–1.22)	0.446
≥27.0	0.40	1.50 (1.03–2.17)	0.034 *
Partner OAB_wet_			
Yes	0.86	2.35 (1.74–3.19)	<0.001 **
Vaginitis			
Yes	0.64	1.89 (1.28–2.80)	0.001 **
Uterine prolapse			
Yes	1.45	4.25 (0.88–20.39)	0.071
Cervical cancer			
Yes	1.69	5.43 (0.57–51.41)	0.140

Ref., Reference group; OAB_wet_, overactive bladder with urinary incontinence. The multivariate logistic regression analysis included variables where *P* < 0.1 in a chi-square analysis of OAB_wet_; these variables were age group, BMI (body mass index), parity, mode of delivery, partner OAB_dry_ (overactive bladder without urinary incontinence) and OAB_wet_, osteoporosis, arthritis, depression, vaginitis, pelvis inflammatory, uterine prolapse, and cervical cancer; * *P* < 0.05; ** *P* < 0.01.

## Data Availability

The data presented in this study are available on request from the corresponding author.
